# Comparative Study between the Surgeon's Intraoperative Evaluation and Histopathology for Diagnosis of Laryngeal Lesions

**DOI:** 10.1155/2014/635251

**Published:** 2014-08-28

**Authors:** Benjamin von Stülpnagel, Robert Hagen, Bernhard Olzowy, Gabriele Witt, Hans Wilhelm Pau, Tino Just

**Affiliations:** Department of Otorhinolaryngology, Head and Neck Surgery, University of Rostock, Doberaner Straße 137-139, 18057 Rostock, Germany

## Abstract

*Objective.* To compare the surgeon's evaluation and histopathology for diagnosis of laryngeal lesions. *Material.* A clinical survey was distributed to laryngeal surgeons, ENT clinicians, and students in 2013 at the Department of Otorhinolaryngology in Rostock. Participants were asked to anonymously identify laryngeal pathologies and to assess the severity of the lesion starting from hyperplasia and inflammation over moderate dysplasia to early laryngeal cancer. Images of similar clinical laryngeal lesions were demonstrated in a multiple-choice modus to assess the surgeon's intraoperative evaluation. The questionnaires were digitally processed and evaluated. The results were correlated with histopathology and compared between experienced laryngeal surgeons, clinicians inexperienced in laryngeal surgery, and medical students from the Medical Faculty of the University of Rostock. *Results.* Sensitivity and specificity varied among the various groups, being highest in experienced laryngeal surgeons. In this group, sensitivity, specificity, positive and negative predictive value, and accuracy were 85%, 56%, 44%, 90%, and 65%, respectively. In 4% and 31%, laryngeal disease was underdiagnosed and overdiagnosed, respectively. In this group, Kappa statistics resulted in Kappa 0.32 (*P* < 0.001). *Conclusion.* This clinical survey clearly demonstrates that conformity between histopathology and evaluation of the laryngeal lesion depends on the surgeon's experience.

## 1. Introduction

Functional outcome and prognosis of laryngeal cancer depend on early diagnosis. Glottic lesions produce very early hoarseness. Standard in diagnosis of early laryngeal cancer is a representative biopsy or excision of the lesion including the basement membrane. Histopathological evaluation of the basement membrane is precondition for assessment of the integrity of this structure and for differentiation between carcinoma in situ and invasive carcinoma of the larynx.

Removal of functional tissue may also produce persistent dysphonia. Therefore, the precision of biopsy or excision of the lesion needs to be as high as possible. With the use of microscope and endoscopes during explorative laryngoscopy, diagnosis of laryngeal cancer improved significantly [1]. However, the diagnosis of laryngeal cancer cannot be proven with the first biopsy in all patients [2], in particular in patients with similar suspicious lesions of the larynx [3].

It is generally accepted that laryngeal cancer derives from dysplasia. The impact of laryngeal dysplasia on the development of laryngeal cancer was assessed in a retrospective study [4]. The authors stated that the risk for development of larynx carcinoma from laryngeal dysplasia revealed no statistical correlation to the initial stage of dysplasia grade. This poses the question of whether the biopsy taken for histopathology was representative for diagnosis. Different entities may be included within a single sample. Rebiopsies for diagnosis of laryngeal cancer may lead to fairly different histopathologies depending on the experience of the surgeon and the size and the side of sampling.

A clinical survey performed a comparison of conformity of the surgeons' assessment of the severity of the laryngeal lesion with histopathology. This survey was distributed to laryngeal surgeons, ENT clinicians, and students in 2013 at the Department of Otorhinolaryngology in Rostock to measure the degree of agreement between the participants' assessment with histopathology.

## 2. Material and Methods

**Figure 1:**
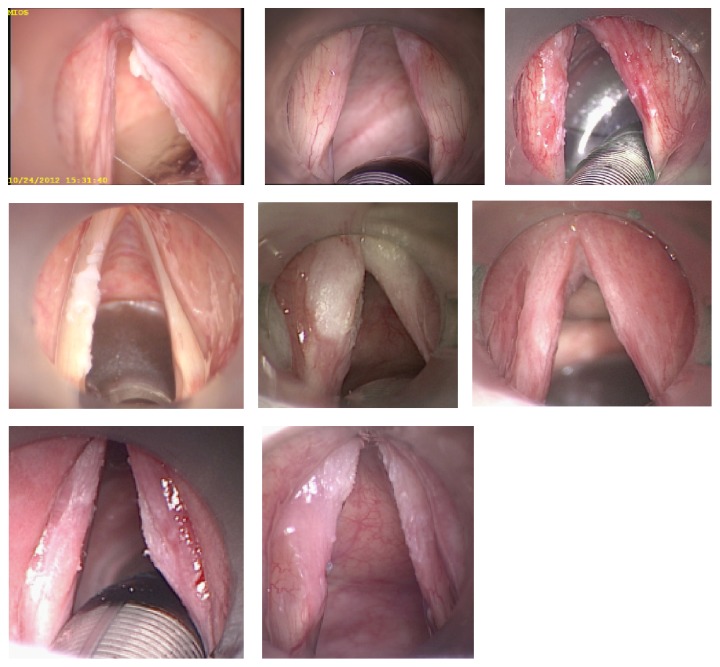
Photographs of laryngeal lesions presented in this survey.

**Figure 2:**
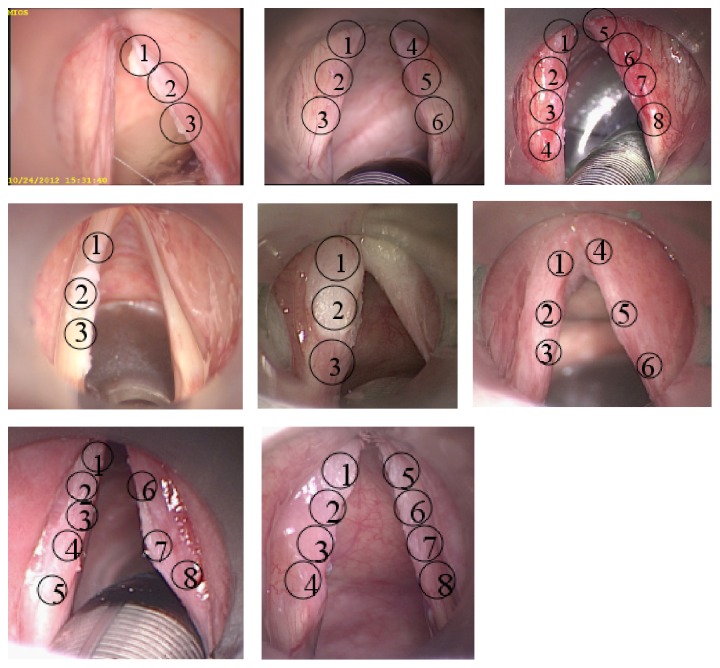
Photographs of laryngeal lesions presented in this survey with marked biopsy regions.

A survey containing 24 questions was distributed among 5 experienced laryngeal surgeons, 6 ENT clinicians (less experienced in laryngeal surgery), and 11 students from the University of Rostock in 2013. An announcement with a short instruction was made asking to participate and to get consent. The survey contained questions about the annual number of surgeries (except students) in laryngeal cancer (microlaryngoscopy and laser surgery). The presentation contained 8 laryngeal images of unilateral or bilateral laryngeal lesions obtained during microlaryngoscopy (Figure 1). Each image was presented three times. Laryngeal lesions selected for this survey showed clinically similar characteristics, which could not be easily assigned as benign or malign. For orientation, all images clearly depicted the true vocal cords. The first slide contained the question of how each participant would diagnose the laryngeal lesion (biopsy, excision, and subepithelial chordectomy). The second slide contained the question of which diagnosis is expected in histopathology (hyperplasia, inflammation, hyperkeratosis, dysplasia (low, moderate, and high grade), carcinoma in situ, and invasive carcinoma). Information about mobility of the epithelium was not presented. The evaluation was based only on microlaryngoscopic outer appearance of the true vocal cords in this question. In the last slide, all participants were asked where a biopsy should be taken. Up to 8 regions of each true vocal cord of each side were indicated by a circle (Figure 2). Multiple biopsies were possible for each case presented in this survey. For each lesion and for each biopsy, all participants had to answer which diagnosis is expected in histopathology (hyperplasia, inflammation, hyperkeratosis, dysplasia (low, moderate, and high grade), carcinoma in situ, and invasive carcinoma).

In 25 out of 45 biopsy regions indicated by circles (3–8 for each patient), a histopathological result was proven. In preparation of this study, the region of the biopsy taken for histopathology was marked during microlaryngoscopy.

For each group, conformity of the surgeons' assessment of the severity of the laryngeal lesion with histopathology (underdiagnosis and overdiagnosis), sensitivity, specificity, positive and negative predictive value, and accuracy were calculated. The degree of conformity was assessed for each biopsy.

*Data Analyses.* For calculation of sensitivity, specificity, positive and negative predictive value, and accuracy, nondysplastic, low grade, and moderate grade dysplasia/squamous intraepithelial neoplasia (SIN) were defined as low grade lesion, while high grade SIN/severe dysplasia, carcinoma in situ, and invasive carcinoma were assigned to high grade lesions. Using Kappa statistics, the agreement between the participants' assessment and histopathology was calculated [5]. Software SPSS 21.0 (SPSS, Inc., Chicago, IL) was used for all calculations, and *P* value of less than 0.05 was considered statistically significant.

## 3. Results

Inconsistencies were found among the three groups. Detailed results are presented in Tables 1 [Table tab2] [Table tab3] [Table tab4] [Table tab5]–6. Laryngeal surgeons tend slightly more than the other clinicians to excise the complete lesions rather than take a biopsy (*P* > 0.05). In the experienced group, laryngeal surgeons indicated in average four biopsies per patient (overall 161 biopsies were indicated including the diagnosis made in the second question of each image) (Table 1).

In terms of agreement of histopathology and assessment of the lesion, assignments by a two-tiered classification (low grade lesion and high grade lesion) resulted in Kappa = 0.32 (*P* < 0.001) for this group. Sensitivity, specificity, positive and negative predictive values, and predictive accuracy were calculated, and these were found to be 85% (95% CI of 71–93%), 56% (95% CI of 46–65%), 44% (95% CI of 34–52%), 90% (95% CI of 80–96%), and 65%, respectively (Table 2). 4% were underdiagnosed, while 31% were overdiagnosed. In the clinician group, the agreement between the clinicians assessment and histopathology resulted in Kappa = 0.262 (*P* < 0.001) (see also Table 3). In terms of the number of biopsies, clinicians decided to take 125 out of 150 possible probes, resulting in a total of 172 diagnoses including those of the second question. Sensitivity, specificity, positive and negative predictive values, and predictive accuracy were 68% (95% CI of 54–79%), 61% (95% CI of 51–70%), 48% (95% CI of 37–59%), 78% (95% CI of 68–86%), and 63%, respectively (Table 4).

In the student group, Kappa statistics revealed Kappa = 0.026 (*P* < 0.001) (Table 5). Sensitivity, specificity, positive and negative predictive values, and predictive accuracy were found to be 48% (95% CI of 39–58%), 61% (95% CI of 55–67%), 39% (95% CI of 31–47%), 70% (95% CI of 63–76%), and 57%, respectively (Table 6).

## 4. Discussion

In this survey, we demonstrated (1) that sensitivity and specificity varied among the various groups being highest in experienced laryngeal surgeons. In this group, sensitivity, specificity, positive and negative predictive value, and accuracy were 85%, 56%, 44%, 90%, and 65%, respectively. (2) Agreement between histopathology and surgeons assessment of the laryngeal lesions was fair (Kappa = 0.32, *P* < 0.001).

It seems to be clear that no one expects from a surgeon to assess the laryngeal lesion in the same way as histopathology does. The surgeon is used to decide how to handle the laryngeal lesion mainly by assessment of the microscopic and endoscopic appearance of the lesion. Manipulation of the epithelium during microlaryngoscopy may provide information about the fixation of the upper layers of the true vocal cord. Knowledge about the tactile feedback may considerably improve the accuracy. In addition, in this survey, only photographs were presented without any knowledge of the case history, voice analyses, and stroboscopic findings. Stroboscopy was thought to detect early laryngeal cancer. This method fails to differentiate clearly between invasive carcinoma of the true vocal cord and intracellular atypia and to determine the penetration depth of the laryngeal cancer [6]. With white light endoscopy, sensitivity, specificity, and accuracy of 73%, 79%, and 77% were reached in identification of precancerous and cancerous laryngeal lesions [7].

This survey aimed to compare the intraoperative assessment of laryngeal lesions in a study environment with histopathology in dependence on the experience of the participants in the field of laryngology. It has been shown that the agreement is highest in the experienced group. However, the results of Kappa analysis in this group revealed only a fair agreement between histopathology and surgeons' assessment. This finding indicates that similar laryngeal lesions cannot be reliably assessed in all cases. Even in the region of biopsy, there seems to be variability in dependence on the experience of the surgeon. The precision of biopsy cannot be assessed in such a survey. But, it is known that within a single probe various entities can be found ranging from hyperplasia, inflammation, hyperkeratosis, dysplasia, and carcinoma in situ to invasive carcinoma. Only a few millimeters away, different histopathological findings may appear without detection of carcinoma. This well-known aspect was supported by Welge-Lüssen et al. in 1996 [2]. Out of 468 patients in a retrospective study, 32 patients required two to six biopsies to confirm the clinically suspected diagnosis. Time delay in diagnosis is closely connected with rebiopsies. The authors found that a time delay of three months in diagnosing cancer did not have a significant influence in prognosis and organ preservation in particular in small lesions that are suspicious of cancer. They concluded to take a rebiopsy as fast as possible to get a proper diagnosis and to preserve the larynx. This poses the question of whether the rebiopsy is representative or not and whether a rebiopsy is generally needed. Again, precision and practical experiences in diagnosis of laryngeal malignancies play an important role. On the other side, rebiopsies and reexcisions may lead to persistent hoarseness. Against this background, an optical biopsy in patients with unclear and suspicious lesions of the larynx may prove to be helpful not only to detect early invasive cancer but also to improve the precision of the intraoperative biopsy. In 2011, the Third Scientific Meeting of Head and Neck Optical Diagnosis Society (HNODS) aimed to enhance interdisciplinary aspects of optical diagnosis and other photodynamic applications [3]. Optical biopsy is defined as the use of light of varying wavelength to assess tissue suspicious for early cancer. The intraoperative use of optical technologies will not replace histopathology. Further clinical research projects will focus on the differentiation between high and low grade lesions of the larynx. Currently, the intraoperative assessment of the suspicious lesion with a microscope and endoscopes and with the involvement of the knowledge of the tactile feedback is the gold standard in laryngeal diseases.

In 2010, 40 ENT surgeons and 40 pathologists attended a national workshop. As a result of this meeting, a consensus paper was published [8]. In presence of widespread laryngeal leukoplakia and confluent leukoplakia, histopathologic mapping of the laryngeal lesion with multiple biopsies was recommended, followed by staged resection if feasible. In addition, it was noted that laryngeal biopsies varied in size and surgeons should try to take larger biopsies when possible to make orientation and evaluation more reliable, particularly if there were previous difficulties in interpretation of the pathology. This poses the question of whether it is feasible to improve the precision and accuracy of biopsy only by training to identify pathological regions within the epithelium and to get a reliable biopsy for diagnosis.

The presented study includes only 5 laryngologists experienced in the field of laryngology. A further study should be designed as a multicenter study including members of the head and neck cancer team rather than general otorhinolaryngologists.

## 5. Conclusion

This clinical survey clearly demonstrates that conformity between histopathology and evaluation of the laryngeal lesion depends on the surgeon's experience. Despite the expertise of experienced laryngeal surgeons, the evaluation of the intraoperative findings does not always correlate with histopathology. Considering the small number of interrogated clinicians, the study reveals a relatively low accuracy of intraoperative assessment of biopsy and excision biopsy in particular for patients with similar suspicious laryngeal lesions and when medical history, stroboscopic findings, and tactile feedback are not presented.

## Figures and Tables

**Table 1:** Comparison of the results between surgeons' assessment and histopathology in the experienced group (laryngeal surgeons).

Histopathology		Surgeons' assessment	
Number		71	90
		Low grade lesion	High grade lesion
114	Low grade lesion	64	50
47	High grade lesion	7	40

**Table 2 tab2:** Agreement of laryngeal surgeons' assessment and histopathology.

	Percentage		Percentage (count)
Sensitivity	85		
Specificity	56	Underdiagnosed	4 (7)
PPW	44	Overdiagnosed	31 (50)
NPW	90	Correct diagnosis	65
Accuracy	65		

PPW: positive predictive value; NPW: negative predictive value.

**Table 3 tab3:** Comparison of the results between surgeons' assessment and histopathology in the clinicians group (clinicians with fewer experiences in laryngeal surgery).

Histopathology		Surgeons' assessment	
Number		88	84
		Low grade lesion	High grade lesion
113	Low grade lesion	69	44
59	High grade lesion	19	40

**Table 4 tab4:** Agreement of clinicians' assessment and histopathology (clinicians with fewer experiences in laryngeal surgery).

	Percentage		Percentage (count)
Sensitivity	68		
Specificity	61	Underdiagnosed	11 (19)
PPW	48	Overdiagnosed	26 (44)
NPW	78	Correct diagnosis	63
Accuracy	63		

PPW: positive predictive value; NPW: negative predictive value.

**Table 5 tab5:** Comparison of the results between students' assessment and histopathology in the student group (inexperienced participants).

Histopathology		Students' assessment	
Number		205	149
		Low grade lesion	High grade lesion
234	Low grade lesion	143	91
120	High grade lesion	62	58

**Table 6:** Agreement of students' assessment and histopathology (inexperienced participants).

	Percentage		Percentage
Sensitivity	48		
Specificity	61	Underdiagnosed	18 (62)
PPW	39	Overdiagnosed	26 (91)
NPW	70	Correct diagnosis	57
Accuracy	57		

PPW: positive predictive value; NPW: negative predictive value.
